# Current Status of Transoral Surgery for Patients With Early-Stage Pharyngeal and Laryngeal Cancers in Japan

**DOI:** 10.3389/fonc.2021.804933

**Published:** 2021-12-14

**Authors:** Daisuke Sano, Akira Shimizu, Ichiro Tateya, Kazunori Fujiwara, Yo Kishimoto, Takashi Maruo, Yasushi Fujimoto, Terushige Mori, Hisayuki Kato, Kiyoaki Tsukahara, Nobuhiko Oridate

**Affiliations:** ^1^Department of Otorhinolaryngology-Head and Neck Surgery, School of Medicine, Yokohama City University, Yokohama, Japan; ^2^Department of Otorhinolaryngology, Head and Neck Surgery, Tokyo Medical University, Tokyo, Japan; ^3^Department of Otolaryngology, Head and Neck Surgery, Fujita Health University, Toyoake, Japan; ^4^Department of Otolaryngology, Head and Neck Surgery, Tottori University, Yonago, Japan; ^5^Department of Otolaryngology-Head and Neck Surgery, Graduate School of Medicine, Kyoto University, Kyoto, Japan; ^6^Department of Otorhinolaryngology, Nagoya University Graduate School of Medicine, Nagoya, Japan; ^7^Department of Otolaryngology, Aichi Medical University, Nagakute, Japan; ^8^Department of Otolaryngology, Head and Neck Surgery, Kagawa University, Kagawa, Japan

**Keywords:** transoral surgery, transoral videolaryngoscopic surgery (TOVS), endoscopic laryngo-pharyngeal surgery (ELPS), transoral robotic surgery (TORS), da Vinci Robotic Surgical System

## Abstract

As the laryngopharynx is closely related to swallowing, speech, and phonation, it is necessary to consider not only disease control but also a minimally invasive approach for the treatment of laryngopharyngeal cancer. Transoral surgery has been reported to be a minimally invasive method for treating these diseases. Transoral videolaryngoscopic surgery (TOVS) and endoscopic laryngo-pharyngeal surgery (ELPS) have been developed in Japan and recently emerged as treatments for patients with early stage pharyngeal and laryngeal cancers. However, securing an appropriate field of view and a narrow operating space during TOVS or ELPS are critical issues to be resolved for these surgeries. The clinical significance and safety of transoral robotic surgery (TORS) using the da Vinci Surgical System have been widely reported to provide surgeons with increased visualization and magnification, resulting in precise surgical margins and rapid functional recovery. In this context, a multi-institutional clinical study was conducted to evaluate the treatment outcomes of TORS for the treatment of laryngopharyngeal cancer in Japan, and the da Vinci Surgical System for oral robot-assisted surgery for these diseases was approved by the Pharmaceutical Affairs Agency in August 2018. This review provides an overview of the therapeutic effects of TOVS, ELPS, and TORS, with a particular focus on these therapeutic results in Japan.

## Introduction

Since the pharynx and larynx play an important role in swallowing, vocalization, and breathing, it is necessary to consider the quality of life (QoL) of patients after treatment for pharyngeal and laryngeal cancer. Although radiation therapy has been widely used as a non-surgical minimally invasive treatment for patients with these diseases, radiation-induced acute adverse events, including dermatitis or mucositis, and late adverse events, such as xerostomia, dysgeusia, hypothyroidism, osteoradionecrosis, and dysphagia, are often irreversible (1). In addition, chemoradiation therapy (CRT) with the addition of cisplatin to radiation, the standard non-surgical curative treatment for advanced pharyngo-laryngeal squamous cell carcinoma (SCC), causes more severe adverse events. Notably, severe late dysphagia after CRT in these patients could be fatal, as suggested by the long-term results of the landmark Radiation Therapy Oncology Group (RTOG) 91-11 study (2). Furthermore, it is necessary to note that salvage surgery for post-irradiation recurrence is associated with an increased risk of complications due to impaired wound healing and the possibility of future development of asynchronous head and neck neoplasms or radiation-induced cancers in young head and neck cancer patients treated with RT or CRT.

In this context, transoral resection as a minimally invasive surgery has been developed for early stage pharyngeal and laryngeal cancer and is widely performed. Transoral surgery aims to shorten the healing time and minimize the loss of function by minimizing the extent of resection without requiring an external incision in the neck. Transoral laser microsurgery (TLM) can be recognized as a milestone of this approach, which was established about 20 years ago by Steiner et al. (3). However, large tumors may require piecemeal resection, as it is sometimes difficult to obtain a sufficient surgical view for TLM, as this surgery is performed under a microscope. In Japan, unique transoral approaches, such as transoral videolaryngoscopic surgery (TOVS) (4) and endoscopic laryngo-pharyngeal surgery (ELPS) (5), have been developed for the surgical treatment of early stage pharyngeal and laryngeal cancers, and are widely used in clinical practice (6). Furthermore, transoral robotic surgery (TORS) has recently emerged as a minimally invasive treatment for pharyngeal and laryngeal cancers, especially in the United States. This approach provides surgeons with a stabilized robotic arm in a precisely magnified field of view, resulting in precise tumor resection (7). Thus, there are several modalities of transoral resection for pharyngeal and laryngeal cancers; however, their therapeutic efficacy has not been sufficiently compared. In this review, we address the current status and therapeutic efficacies of transoral surgeries for patients with early stage pharyngeal and laryngeal cancers, including TOVS, ELPS, and TORS.

## TOVS in Japan

**Figure 1:**
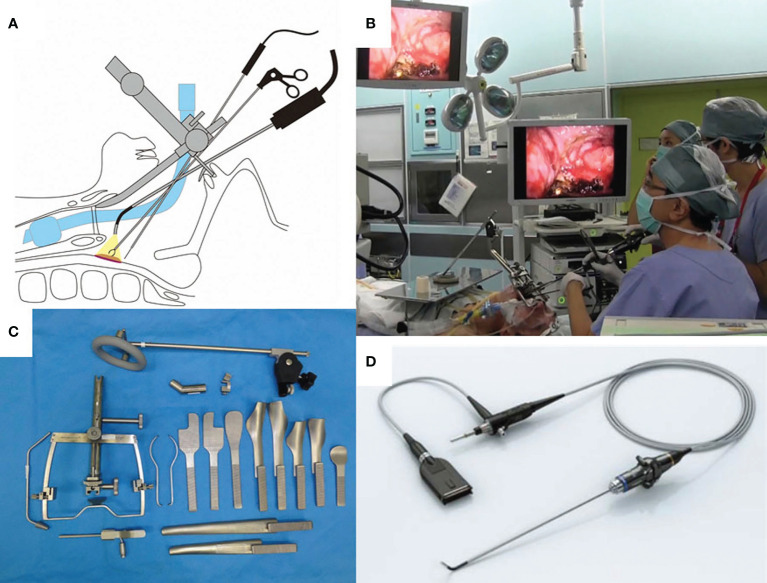
**(A)** Shema of transoral videolaryngoscopic surgery (TOVS). **(B)** Surgical setup of TOVS. **(C)** FK-WO retractor (Olympus Medical Systems, Tokyo Japan). **(D)** Endoeye Flex (Olympus). Used with permission from (6).

TOVS is a minimally invasive transoral resection technique developed by Shiotani et al. (4). In TOVS, the field of view is secured by a Weerda-type distending laryngoscope (Karl Storz, Tuttlingen, Germany), FK-WO retractor (Olympus Medical Systems, Tokyo, Japan), or Davis-type mouth gag depending on the primary site, and the surgical field is visualized on a high-definition video monitor with an inserted rigid endoscope, such as Endoeye Flex (Olympus Medical Systems), through the side of the laryngoscope (**Figure 1**). Thus, the ultra-wide-angle lens attached to the tip of the endoscope allows for a wider direct field of surgical view than that visible under a microscope. In addition, tumors can be resected through bimanual manipulation of existing straight forceps, ultrafine electrocautery, and hemostatic instruments, using the direct technique that otolaryngologists have traditionally mastered (4). Another advantage of the TOVS system is the use of a universal endoscope system for this procedure, for example, the narrow band imaging (NBI) system (Olympus), which is commonly used to determine early stage cancer in upper gastrointestinal endoscopy, is also available for this procedure when observing lesions, resulting in accurate observation of the extent of lesion growth, including submucosal lesions. Transoral resection is commonly performed with an electrosurgical needle knife, and hemostasis is achieved with suction cautery (Karl Storz) or hemoclips (Karl Storz). TOVS is usually indicated for Tis lesions, including superficial Tis and T1–2 lesions that are subject to conventional open surgery, while laryngeal T3 lesions without deep tissue involvement may also be suitable in some cases.

Thus, TOVS has many advantages as a minimally invasive surgery; however, there have only been a few reports on treatment outcomes and safety (8, 9). Recently, Tomifuji et al. reported the long-term treatment outcomes of 90 patients with hypopharyngeal cancer and 25 patients with supraglottic cancer who underwent TOVS, including advanced T3 or T4 lesions treated with neoadjuvant chemotherapy or before surgery or cases of failed radiation therapy, in their single institutional retrospective observational study (10). A total of 83 patients with fresh hypopharyngeal cancer (6 with Tis, 18 with T1, 45 with T2, 12 with T3, and 2 with T4) had a 5-year overall survival (OS) rate of 83.2%, 5-year disease-specific survival (DSS) rate of 94.3%, and 5-year larynx preservation rate (LPR) of 94.6%. Twenty patients with fresh supraglottic cancer (7 cases of T1 and 13 cases of T2) had a 5-year OS rate of 80.0%, DSS rate of 95%, and LPR of 94.7%. Of the 115 patients who underwent TOVS, three (2.6%) had postoperative hemorrhage, four (3.4%) underwent emergency tracheostomy, and 11 (9.5%) in total underwent tracheostomy. Permanent tracheostomy was required in four cases (3.4%). Postoperative swallowing function was generally good in their study; however, oral intake was abandoned in four patients within six months after TOVS. In fact, two patients were tube-feeding-dependent, one patient required total laryngopharyngectomy due to severe postoperative stenosis of the pharynx, and one patient required laryngotracheal separation due to severe swallowing dysfunction (**Table 1**). To date, there are no reports comparing the treatment outcomes of TOVS with those of other modalities.

**Table 1:** Treatment outcomes of TOVs, ELPs, and TORS.

Source	Study design	No. of Primary sites					Treatment outcomes	Positive margins	Post Operative (C) RT	Complications
		control	patients	Hypopharynx	Oropharynx	Supraglottic				
**TOVS**										
Tomifuji et al (8)	retrospective	NA	60	30	18	12	5-yrs OS: 77%	Positive: 5	RT: 17, CRT: 7	Postoperative bleeding: 2
							5-yrs DSS: 95%			Temoral emphysema: 3
Imanishi et al (9)	retrospective	NA	72	58	0	14	5-yrs OS: 77.9%	very close to or an equivocal surgical margin: 3	CRT: 12	Tracheostomy in total: 2
							5-yrs CSS: 87.3%			
Tomifuji et al (10)	retrospective	NA	115	90	0	25	5-yrs OS: 83.2%*	NA	RT: 20, CRT: 8	Tracheostomy in total: 11
							5-yrs DSS: 94.3%*			Postoperative bleeding: 3
							5-yrs LPR: 94.6%*			Permanent tracheostomy: 3
										Tube feeding dependency: 2
**ELPS**										
Satou et al (11)	retrospective	NA	113	173	0	0	5-yrs OS: 45.2%	NA	NA	Tracheostomy in total: 13
							5-yrs DSS: 87.5%			Postoperative bleeding: 4
Tateya et al (12)	retrospective	NA	75	74	28	2	3-yrs OS: 90%		None	Postoperative bleeding: 3
							3-yrs DSS: 100%			Temoral emphysema: 10
Kishimoto et al (13)	retrospective	NA	22**	17	12		3-yrs OS: 90.2%	Positive: 4	None	Postoperative bleeding: 2
							3-yrs DSS: 100%			Aspiration pneumonia: 2
**TORS *vs.* non-robotic**										
Chen al (14)	retrospective	Yes								
		TORS	877	877	0	0	NA	Positive: 170	RT: 216, CRT: 302	
		non-robotic	4269	4269	0	0	NA	Positive: 1157	RT: 668, CRT: 1953	
Richmon al (15)	retrospective	Yes								
		TORS	116	116	0	0	NA	NA	NA	Tracheostomy in total: 0
		non-robotic	9485	9485	0	0	NA	NA	NA	Tube feeding dependency: 0
Motz et al (16)	retrospective	Yes								
		TORS	304	304	0	0	NA	NA	RT: 33%, CRT: 33.3%	tracheotomy during treatment: 3.9% (*vs.* 11.4% in non-robotic)
		non-robotic	3268	3268	0	0	NA	NA	RT: 25%, CRT: 39.8%	Posttreatment gastrostomy tube use: 21.9% (*vs.* 34.2% in non-robotic)
Chillakuru (17)	retrospective	Yes								
		TORS	2288	2288	0	0	HPV+ 5-yrs OS: 91.2% (stage I), 81.2 (stage II), 53.5 (stage III).	Positive: 235, Missing: 29	RT: 606, CRT: 600, Missing: 14	NA
							HPV- 5-yrs OS: 82.6% (stage I), 80.4% (stage II), 75.6% (stage III),			
							66.6% (stage IV)			
		non-robotic	3167	3167	0	0	HPV+ 5-yrs OS: 87.0% (stage I), 73.2 (stage II), 71.1 (stage III).	Positive: 640 Missing: 118	RT: 558, CRT: 1091, Missing: 32	NA
							HPV- 5-yrs OS: 66.8% (stage I), 61.8% (stage II), 68.8% (stage III),			
							61.8% (stage IV)			
Sano et al (18)	retrospective	Yes								
		TORS	68	57	10	1	NA	Positive: 7, unknown: 3	CRT: 2	NA
		non-robotic	236	73	154	9	NA	Positive: 57, unknown: 12	RT: 6, CRT: 3, missing data: 1	NA
		HNCRJ	1228	969	171	88	NA	NA	RT: 47, CRT: 36, missing data: 4	NA

*in patients with fresh hypopharyngeal cancer. **patients aged 75 years or older. NA, not available.

## ELPS in Japan

**Figure 2:**
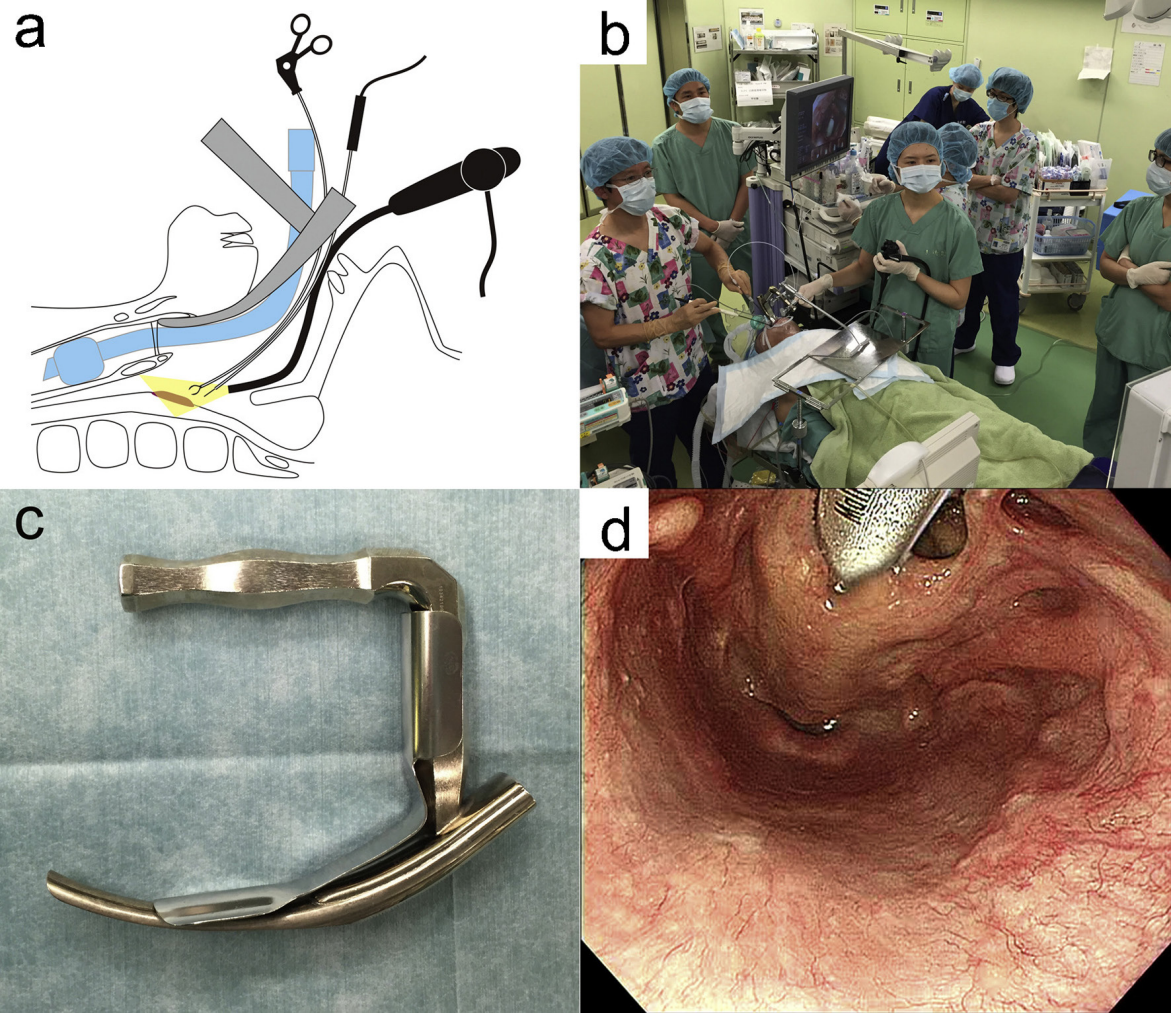
**(A)** Shema of endoscopic laryngo-pharyngeal surgery (ELPS). **(B)** Surgical setup of ELPS. **(C)** Rigid curved larygo-pharyngoscope (Nagashima Medical Instruments, Tokyo, Japan). **(D)** Surgical view of ELPS by the rigid curved larygo-pharyngoscope. Used with permission from (6).

**Figure 3:**
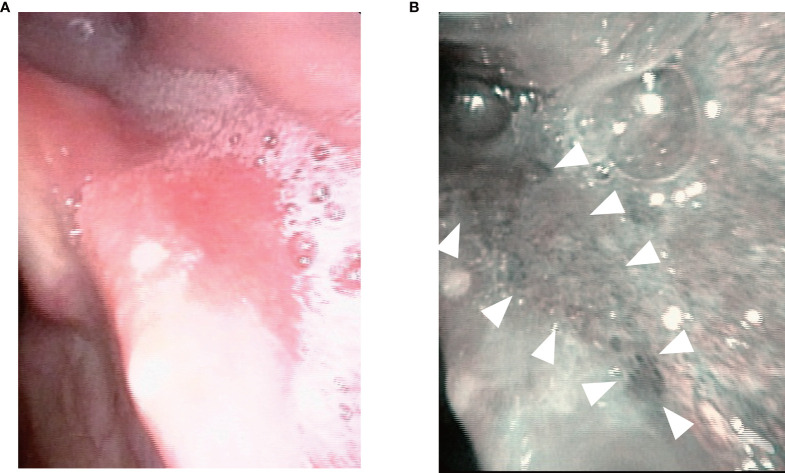
Endoscopic imaging of a small submucosal lesion in the left piriform sinus. **(A)** The lesion was not clearly visualized with normal white light. **(B)** narrow band imaging enhanced a mucosal lesion with brownish area or scattered brown dots (arrowhead).

The TOVS procedure was established to overcome the disadvantages of TLM, whereas ELPS was developed based on the endoscopic submucosal dissection (ESD) procedure for early esophageal cancer to resect superficial mucosal lesions of the pharynx or larynx transorally developed by Satou et al. (5). In ELPS, the field of view is normally secured using a curved rigid laryngoscope (Nagashima Medical Instruments Company, Ltd., Tokyo, Japan), and the surgical field is visualized using a flexible endoscope inserted transorally by a gastroenterologist or assistant of head and neck surgeon. In this procedure, tumor resection is often performed by a head and neck surgeon using specially designed curved forceps (Nagashima Medical Instruments Company, Ltd.) and a curved electrosurgical needle (Olympus Medical Systems, Tokyo, Japan) with subepithelial injection to elevate the tumor from the submucosal tissue (**Figure 2**). In some cases, the gastroenterologist who inserts the flexible fiberesophagoscope performs tumor resection in the manner of ESD (19). This curved laryngoscope enables clinicians to obtain a view of the entire hypopharynx from the tip of the pyriform sinus to the post-cricoid area and the entrance of the esophagus, and to accurately identify the extent of submucosal lesions using NBI (12). ELPS, which was established based on the ESD procedure, has advantages as a minimally invasive transoral resection of early stage or submucosal lesions of hypopharynx. NBI can clearly depict the mucosal surface microstructure. Abnormal growth of microvessels, described as brownish area or scattered brown dots, has been recognized as a characteristic finding of early-stage upper gastrointestinal cancer (**Figure 3**), and thus NBI is useful in identifying these submucosal lesions. ELPS emerged nationwide in clinical practice in Japan; however, there have been few reports on its treatment outcomes and safety. Satou et al. reported the long-term treatment outcomes of 177 fresh superficial laryngopharyngeal cancer lesions in 113 patients and five patients with residual or recurrent disease after irradiation underwent ELPS in their single institutional retrospective observational study (11). The patients with a total of 177 fresh superficial laryngopharyngeal cancer lesions (23, superficial-type; 53, superficial elevated-type or superficial elevated + flat-type; 92, superficial flat type; 8, superficial depressed or superficial depressed + elevated-type or superficial depressed + flat-type, and 1, excavated-type) had a 5-year OS rate of 45.2% and a 5-year DSS rate of 87.5. Out of the 118 patients, four (3.4%) had postoperative hemorrhage, 13 (11.0%) underwent tracheostomy postoperatively, and one patient required pharyngeal reconstruction with laryngotracheal separation with pectoral major musculocutaneous flap due to severe postoperative stenosis of the pharynx (**Table 1**). Tateya et al. also retrospectively evaluated the treatment outcomes of 75 consecutive patients with 104 fresh superficial laryngopharyngeal cancers, reporting a 3-year OS rate of 90.0% and 3-year DSS rate of 100% (12). Of the 75 patients, three (4.0%) had postoperative hemorrhage in their study. Kishimoto et al. reported the treatment outcomes of 29 cancerous or precancerous lesions in 19 patients aged ≥75 years who underwent ELPS with a 3-year OS rate of 90.2% and 3-year DSS rate of 100%. In their cohort, two patients had postoperative hemorrhage, and two patients had aspiration pneumonia (13). A mutant allele encoding an inactive subunit of aldehyde dehydrogenase-2 is reported to be frequently observed in the Japanese population (20), resulting in their high risk for malignancies of the upper gastrointestinal tract. As screening by upper gastrointestinal endoscopy is relatively widespread in Japan, there may be a small number of studies on early-stage hypopharyngeal cancer as described above. To date, there are no reports comparing the treatment outcomes of ELPS with those of other modalities.

## Treatment Outcomes of TORS

TORS was established in 2005 since Weinstein and O’Malley et al. installed the da Vinci Robotic Surgical System (Intuitive Surgical, Sunnyvale, CA) for transoral surgery (21, 22). The stabilized robotic arms used and clear surgical view with high-definition three-dimensional endoscopy imaging in TORS allow surgeons to resect tumors precisely. In fact, a multicenter clinical trial of TORS mainly for T1/T2 oropharyngeal carcinoma (combined neck dissection if node-positive) reported excellent results with a 2-year local control rate of 91.8%, DSS rate of 94.5%, and OS rate of 91.0% (23). In a total of 177 patients in a multicenter clinical trial, Weinstein et al. also reported good treatment outcomes of TORS with a surgical positive margin of 4.3% and good postoperative functional preservation with a gastrostomy dependency rate of 5.0% and a permanent tracheostomy residual rate of 2.3% (24). Thus, the clinical significance and safety of TORS for head and neck cancer, particularly for oropharyngeal squamous cell carcinoma (OPSCC), have been widely reported in the past decade (23, 25, 26).

In recent years, big data analysis using nationwide databases has been conducted to compare treatment outcomes between TORS and conventional surgical resection. Richmon et al. reported the results of a retrospective cross-sectional study using discharge data from a nationwide inpatient sample of 116 patients who underwent TORS and 9485 patients who underwent non-robotic surgery between 2008 and 2009. They reported that TORS was associated with a lower postoperative gastrostomy dependency rate, tracheostomy placement rate, and post-treatment unplanned hospitalization rate than non-robotic surgery, and enabled shorter hospital stays, resulting in reduced costs of care (15). Chen et al. reported the results of a retrospective database review using the National Cancer Database with 877 patients who underwent TORS and 4269 patients who underwent non-robotic surgery between 2010 and 2011. In their analysis, TORS showed a significantly lower rate of surgical positive margins than nonrobotic surgery (14). Mots et al. also reported the results of a retrospective cross-sectional analysis of 3573 oropharyngeal cancer (OPC) patients (304 patients who underwent TORS and 3268 patients who underwent non-robotic surgery) from 2010 to 2012 using the MarketScan Commercial Claim and Encounters database. They showed that TORS had a lower rate of tracheotomy during treatment (3.9% *vs.* 11.4%) and post-treatment gastrostomy tube use (21.9% *vs.* 34.2%) than non-robotic surgery (16). Furthermore, a retrospective cohort study using de-identified data from the National Cancer Database from 2010 to 2016 by Chillakuru et al., comparing TORS (N=2288) with non-robotic surgery (N=3167), showed that patients who underwent TORS had better OS rates than those who underwent non-robotic surgery regardless of human papillomavirus status (17). After TORS using the da Vinci Robotic Surgical System (Intuitive Surgical) was approved by the United States Food and Drug Administration in 2009, TORS has now spread nationwide for the treatment of pharyngeal and laryngeal cancer in the United States. In fact, the treatment statistics for T1–T2 OPC lesions in the National Cancer Database showed a dramatic increase from 56% for surgery in 2004 to 82% in 2013 (27).

Systematic reviews and meta-analyses of the treatment outcomes of TORS are also beginning to emerge. Castellano et al. systematically reviewed the post-treatment QoL and swallowing function of 659 patients with head and neck cancer who underwent TORS. Several studies have shown better postoperative QoL and swallowing function with TORS than with open surgery or CRT (28). Park et al. reported the results of their systematic review and meta-analysis including nine non-randomized studies to compare the safety and effectiveness of TORS and open surgery for OPC (29). In their study, TORS had better a disease-free survival rate and lower risk of free flap reconstruction than those of open surgery. Stokes et al. performed a systematic review and meta-analysis to present low post-TORS hemorrhage (5.8%), including major hemorrhage requiring emergent embolization, transcervical arterial ligation, or tracheotomy (2.9%) (30).

Although there are more than 160 da Vinci surgical systems in Japan, the Japanese FDA has not yet approved TORS for the treatment of head and neck cancer, leading Japanese doctors to develop their own transoral approaches, such as TOVS and ELPS, as mentioned above (6). In August 2018, the da Vinci Robotic Surgical System (Intuitive Surgical) for TORS was finally approved by the Pharmaceutical Affairs Agency as a result of a multicenter clinical trial conducted by Tottori University, Kyoto University, and Tokyo Medical University under the Advanced Medical Care B program in Japan. TORS is now available nationwide as a transoral approach for the treatment of head and neck cancer, although it is not covered by Japanese public insurance (18).

## Treatment Outcomes of TOVS, ELPS, and TORS in Japan

Thus, TORS has recently been established in Japan; however, there have been no studies comparing its treatment outcomes and safety with other transoral surgeries established in Japan, such as TOVS and ELPS. In the context of this background, Sano et al. retrospectively evaluated the treatment efficacy and subsequent postoperative treatment of TORS to compare with those of any non-robotic transoral surgery, including TOVS, ELPS, and TLM, in patients with laryngeal and pharyngeal SCC (18). They used data from patients who were assumed to have undergone non-robotic transoral surgery, collected from the Head and Neck Cancer Registry of Japan, to validate this comparison. The main endpoints of this multicenter retrospective observational study were the presence of positive surgical margins and the requirement for postoperative treatment. Patients who underwent concurrent neck dissection or cervical lymph node metastasis were excluded from this study. A total of 68 patients who underwent TORS (the TORS cohort), 236 patients who underwent non-robotic transoral surgery (the non-robotic cohort), and 1228 patients collected from the Head and Neck Cancer Registry of Japan (the registry cohort) were eligible for this study. In the comparison of the overall population, the proportional distribution of the primary site of disease was found to vary between cohorts, with patients in the TORS cohort having a higher proportion of oropharyngeal tumor disease than other diseases. In addition, the TORS cohort included more patients with advanced disease than the other cohorts and showed a lower rate of positive surgical margin than that of the non-robotic cohort (10.3% *vs.* 24.2%, *P*=0.018). The TORS cohort also showed a lower rate of postoperative treatment than that of the other cohorts (2.9% *vs.* 4.2% [non-robotic cohort] *vs.* 7.1% [registry cohort]).

A total of 57 patients in the TORS cohort, 73 in the non-robotic cohort, and 171 in the registry cohort were eligible for subgroup analysis of patients with OPSCC. The TORS cohort included more lateral wall lesions with more advanced disease than the other cohorts. In the OPSCC population, the TORS showed a lower rate of positive surgical margin than the non-robotic cohort (8.8% *vs.* 24.7%, *P*=0.026) without any postoperative treatment (0.0% *vs.* 5.5% [non-robotic surgery] *vs.* 5.8% [registry cohort]). In this study, the distribution of subsite and T classification of disease, and the rate of postoperative treatment were similar in the non-robotic cohort and registry cohort, suggesting the validity of the non-robotic cohort as a historical control. Although this study had many biases that could not be ruled out due to its design not including any details of surgery-related complications or long-term observations, the results suggested that TORS may be less likely than other non-robotic transoral surgeries to lead to a positive resection margin.

As **Table 1**, which summarizes the previous literature for treatment outcomes of transoral surgeries, shows, TORS may reduce the rate of positive surgical margins relative to that with non-robotic surgery in the treatment of patients with pharyngeal and laryngeal cancer. It should be noted, however, that there have been no well-designed studies to evaluate the treatment outcomes and safety of TORS compared with those of other transoral surgeries. Hence, the superiority of TORS needs to be validated through clinical studies designed to minimize bias in the future.

Currently, all indications for TORS in Japan are managed by the Japanese Robot-Assisted Surgery Committee of the Japan Society of Head and Neck Surgery with an all-case surveillance to evaluate treatment outcomes and safety of TORS (18), which may provide novel real-world data for TORS.

## Conclusion

In this review, we described the current status and treatment outcomes of transoral surgeries, including TOVS, ELPS, and TORS, with a particular focus on these therapeutic results in Japan. As a result of the delay in the introduction of TORS, unique transoral surgical techniques, such as TOVS and ELPS, have been developed and are widely used in Japan. The advantages of TOVS and ELPS are that both can be performed using existing universal surgical instruments and endoscope systems, thereby lowering the cost. As of October 2021, the da Vinci Surgical System for TORS is still not covered by Japanese public insurance, resulting in an insufficient number of patients with head and neck cancers undergoing TORS, although it was approved by the Pharmaceutical Affairs Agency in August 2018. Further investigation to evaluate the treatment outcomes, safety, and cost-effectiveness of TORS compared to those of other transoral surgical approaches, including TOVS and ELPS, is needed in the future. Ongoing all-case surveillance of patients undergoing TORS in Japan could provide novel real-world data regarding treatment with TORS.

## Author Contributions

DS, AS, IT, KF, YK, TaM, YF, KT, TeM, HK, and NO were involved in data acquisition. The first draft of the manuscript was written by the DS and NO. All authors reviewed and commented on the subsequent drafts of the manuscript. All authors contributed to the article and approved the submitted version.

## Conflict of Interest

The authors declare that the research was conducted in the absence of any commercial or financial relationships that could be construed as a potential conflict of interest.

## Publisher’s Note

All claims expressed in this article are solely those of the authors and do not necessarily represent those of their affiliated organizations, or those of the publisher, the editors and the reviewers. Any product that may be evaluated in this article, or claim that may be made by its manufacturer, is not guaranteed or endorsed by the publisher.
